# Structural Insights into Cellulose-Coated Oil in Water Emulsions

**DOI:** 10.1021/acs.langmuir.2c00947

**Published:** 2022-09-07

**Authors:** Ester Korkus Hamal, Gilad Alfassi, Rafail Khalfin, Dmitry M. Rein, Yachin Cohen

**Affiliations:** † Department of Chemical Engineering, Technion—Israel Institute of Technology, Haifa 3200003, Israel; ‡ Department of Biotechnology Engineering, ORT Braude College, Karmiel 2161002, Israel

## Abstract

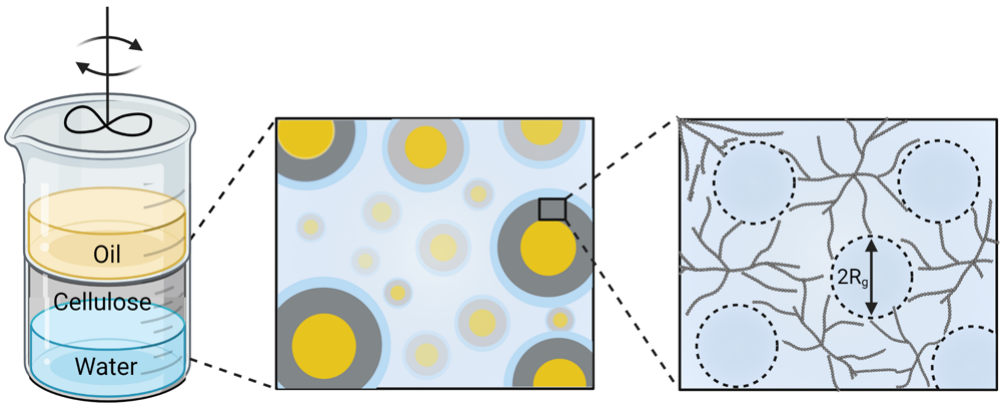

Cellulose is a renewable biopolymer, abundant on Earth, with a multi-level supramolecular structure. There has been significant interest and advancement in utilizing natural cellulose to stabilize emulsions. In our research, we develop and examine oil in water emulsions surrounded by unmodified cellulose as microreactors for the process of transformation of cellulose into valuable chemicals such as biodiesel. This study presents morphological characterization of cellulose-coated emulsions that can be used for such purposes. Cryogenic-scanning electron microscopy imaging along with light microscopy and light scattering reveals a multi-layer inner structure: an oil core surrounded by a porous cellulose hydrogel shell, coated by an outer shell of regenerated cellulose. Measurements of small-angle X-ray scattering provide quantification of the nano-scale structure within the porous cellulose hydrogel inner shell of the emulsion particle. These characteristics are relevant to utilization of cellulose-coated emulsions in various applications such as controlled release and as hosts for enzymatic biotechnological reactions.

## Introduction

In the common global concern, engineering an innovative process for transformation of cellulose into biofuel is a significant goal. Cellulose has unique properties such as low cost, high strength, thermal and chemical stability, non-toxicity, biodegradability, biocompatibility, amphiphilicity, and renewability.^1−3^ The amphiphilic character of the cellulose chains has gained remarkable interest in the implementation of emulsion stabilization.^4−12^ Recently, emulsions were examined and suggested as a novel biphasic system that advances the contact between reagents and simplifies product separation in a biphasic reaction. Moreover, emulsions are applied as encapsulation and delivery systems in pharmaceutical, food, and personal care applications and to protect and enable controlled release of hydrophilic or lipophilic active molecules.^13−15^

Emulsions consist of at least two immiscible liquids, whereby one of the liquids is dispersed as droplets in a continuous phase of the other liquid. O/w emulsions are oil droplets dispersed in an aqueous medium and vice versa.^16,17^ Conventional emulsions are thermodynamically unstable systems; therefore, a third component is required. Emulsions are stabilized using amphiphilic and surface-active molecules. The surfactants adsorb at the oil-water interface, thus preventing emulsion instability due to coalescence, by diverse mechanisms such as electrostatic repulsion of charged units or steric hindrance due to long polymeric chains.^9,18,19^

An early study performed by Pickering demonstrated the ability of solid particles to stabilize emulsion droplets, known as Pickering emulsions. Such systems improve the reaction efficiency, selectivity, and simplify product separation, compared to conventional biphasic systems due to the large reaction interfacial area.^20^ Cellulose nanofibrillar particles, especially nanocrystals, stabilize o/w emulsions without additional surfactants by the Pickering mechanism.^21,22^ Several researchers examined cellulose-stabilized emulsion systems, suggesting that different cellulose crystal surfaces can interact either with water or oil, thus stabilizing o/w emulsions.^4,5^

Cellulose derivatives, designed to exhibit solubility, amphiphilicity, and stabilization functionality by one or more of the known mechanisms, are widely used emulsion stabilizers.^23^ Until quite recently, natural unmodified cellulose has not been applied due to the lack of a suitable processing method for its dissolution. Recent research demonstrated that unmodified cellulose can molecularly stabilize o/w emulsions through the formation of a stable coating from natural cellulose dissolved in an ionic liquid solution through a dissolution and regeneration process.^10^

Since a simple encapsulation process utilizing natural cellulose for engineering multi-functionality is lacking, there is particular focus on engineering more complex systems of encapsulated materials, such as multi-compartment or multi-layered microcapsules. Napso et el. analyzed the structure of cellulose-coated o/w emulsion droplets fabricated from cellulose dissolved in an ionic liquid. The results exhibit a unique internal multilayer structure of the cellulose-coated o/w emulsion particles: an inner oil core, surrounded by a shell composed of a porous cellulose hydrogel with a cellulose volume fraction of ∼3%, encapsulated by a denser external shell of amorphous cellulose with a cellulose volume fraction of ∼25%.^6^ Furthermore, the structure of hydrogels regenerated from the ionic-liquid solutions was related to the solution concentration and the coagulation process.^24^ For practical application, the use of ionic liquids may still be prohibitive. Alfassi et al. reported the fabrication of stable o/w emulsions using a suspension of cellulose hydrogel particles fabricated from cellulose dissolved in cold aqueous NaOH solution and their utilization in enzymatic cellulose hydrolysis.^7^ Nevertheless, further research is needed to assess the formation and structure of such multi-layer cellulose-coated o/w emulsion droplets. In this study, the morphology and the unique multi-layer structure of cellulose-coated o/w emulsion particles, fabricated using hydrogel particles formed by regeneration of cellulose dissolved in cold aqueous NaOH solution, was examined using different hydrophobic phases in order to correlate the structural characteristics of the emulsions to the processing conditions of their formation. Of particular interest are characteristics of the cellulose coating shells: thickness, composition, and porosity. Characterization of the emulsion particles is achieved by imaging using cryogenic-scanning electron microscopy (cryo-SEM), light microscopy, and light scattering (LS), along with small-angle X-ray scattering (SAXS) measurements, which provide a more quantitative evaluation. This research may provide an enhanced understanding of effective utility of such emulsion particles as micro-bioreactors for transformation of cellulose into biodiesel.

## Experimental Section

### Materials

Microcrystalline cellulose (MCC) powder was purchased from Sigma-Aldrich (Israel) (particle size in the range 20–160 μm; degree of polymerization ∼295, as given by the supplier). Sodium hydroxide, *n*-decane, and *n*-hexadecane were obtained from Merck Chemicals (Israel). Castor oil was provided by Chen Samuel chemicals (Israel). Canola oil was obtained from a local supplier.

## Methods

### Emulsion Preparation

MCC powder was dissolved in aqueous NaOH (7 wt %) by mixing at room temperature for 10 min, followed by cooling in an ethylene glycol bath (−16 °C) for 10 min, using a mechanical stirrer 500 rpm, until no visual indication of crystals could be observed. Coagulation of the cellulose solution was obtained by addition of deionized water (without stirring). The coagulated hydrogel was rinsed several times with deionized water for removal of alkali traces, as indicated by electrical conductivity below 1 mS cm^–1^. The cellulose content in the hydrogel was determined gravimetrically (in triplicate).

Cellulose-coated o/w emulsions were obtained by two stages. A pre-emulsion dispersion was first prepared by mixing the cellulose hydrogel dispersion (2.7 wt %), deionized water, and oil (*n*-decane, *n*-hexadecane, castor oil, or canola oil) using the mechanical homogenizer IKA T-18 Ultra-Turrax (IKA Works Inc., USA) for 10 min at 20,000 rpm. The cellulose content in the cellulose-coated o/w emulsions was 1 wt %. High-pressure homogenization (HPH) was applied to the coarse emulsion (microfluidizer Model LM-20, Microfluidics, USA), at different homogenization pressures (14 and 70 MPa) for 4 min. During HPH, the temperature was kept around 40 °C by using ice. The emulsions are designated as: cellulose/oil wt ratio, core liquid, HPH (pressure psi). The hydrogel samples have the same cellulose content as the emulsions (1 wt %).

### Characterization

#### Microscopy

Light microscope images were obtained using the Olympus BH2 light microscope (Olympus, Tokyo, Japan), with a 12-bit cooled CCD camera, using Achromat positive low phase contrast objectives. The imageJ software, scientific image analysis software, was used for image analysis.

The morphology of cellulose-coated o/w emulsion particles was investigated using the Zeiss Ultra Plus high-resolution cryogenic scanning electron microscope. It is equipped with a Schottky field-emission gun and utilizes the Bal-Tec VCT100 (Leica) cold stage maintained at temperatures below −145 °C. Specimen preparation for cryo-imaging involved placing of an emulsion droplet on a stub and vitrification by plunging into supercooled liquid ethane and subsequently into liquid nitrogen. Following transfer to a freeze fracture unit (BAF060), via a pumped cryo-transfer shuttle, maintained at −170 °C, the sample was fractured rapidly by a cooled knife. The cryo-transfer shuttle was again used to transfer the fractured sample to the high-resolution scanning electron microscope for imaging. Temperature was raised to −100 °C for 30 s to remove some of the ice by sublimation to expose structural features and improve contrast.^25^ To minimize charging, the samples were imaged at low electron acceleration voltage (1–1.4 kV). The working distance was 3–4.5 mm. An Everhart–Thornley detector (SE2) was used, providing secondary electron emission contrast due to the surface structure. The images were examined with imageJ software, scientific image analysis software.

#### Light Scattering

Dimensions of emulsified droplets and their size distribution were monitored by LS using the Mastersizer 2000 (Malvern Co. Ltd., UK), equipped with a He–Ne red laser (λ = 633 nm). Volume-based calculations of the droplet size distribution was employed. The measurement was performed in triplicate.

#### Small Angle X-ray Scattering

SAXS measurements were done by the Rigaku SAXS/WAXS instrument (MicroMax −002 + S), including a generator powered at 45 kV and 0.9 mA with a sealed microfocus tube (Cu Kα radiation, λ = 0.154 nm), and collimation by two Göbel mirrors and three pinholes. A two-dimensional position sensitive wire detector (Gabriel) was used to record the scattering patterns, at a distance of 150 cm behind the sample. This provided measurement of the scattering intensity *I*(*q*), as a function of the scattering vector (*q*), where *q*= (4π/λ)sin θ, 2θ is the scattering angle, and λ is the radiation wavelength in the range of 7.5 × 10^–3^ <*q* < 0.264 Å^–1^. Cellulose-coated o/w emulsions were sealed in glass capillaries and measured at 22 °C under vacuum. Scattering from an empty capillary and electronic background was subtracted from the measured intensity. Normalization of the data to the absolute scattering cross section (cm^–1^) was done using pre-calibrated glassy carbon as a secondary calibration standard. Analysis was done using the SasView program.^26^

## Results and Discussion

The morphology and the unique multi-layer structure of oil in water (o/w) emulsions coated by unmodified cellulose was examined for four kinds of emulsions using different hydrophobic phases: *n*-decane, *n*-hexadecane, canola oil, and castor oil. Each kind of emulsion includes two cellulose/oil weight ratios: 1:1 and 1:4. Cryo-SEM imaging, light microscopy and LS characterize the oil emulsion particles coated by layers of cellulose, complemented by SAXS measurements, which provide some more quantitative details.

**Figure 1:**
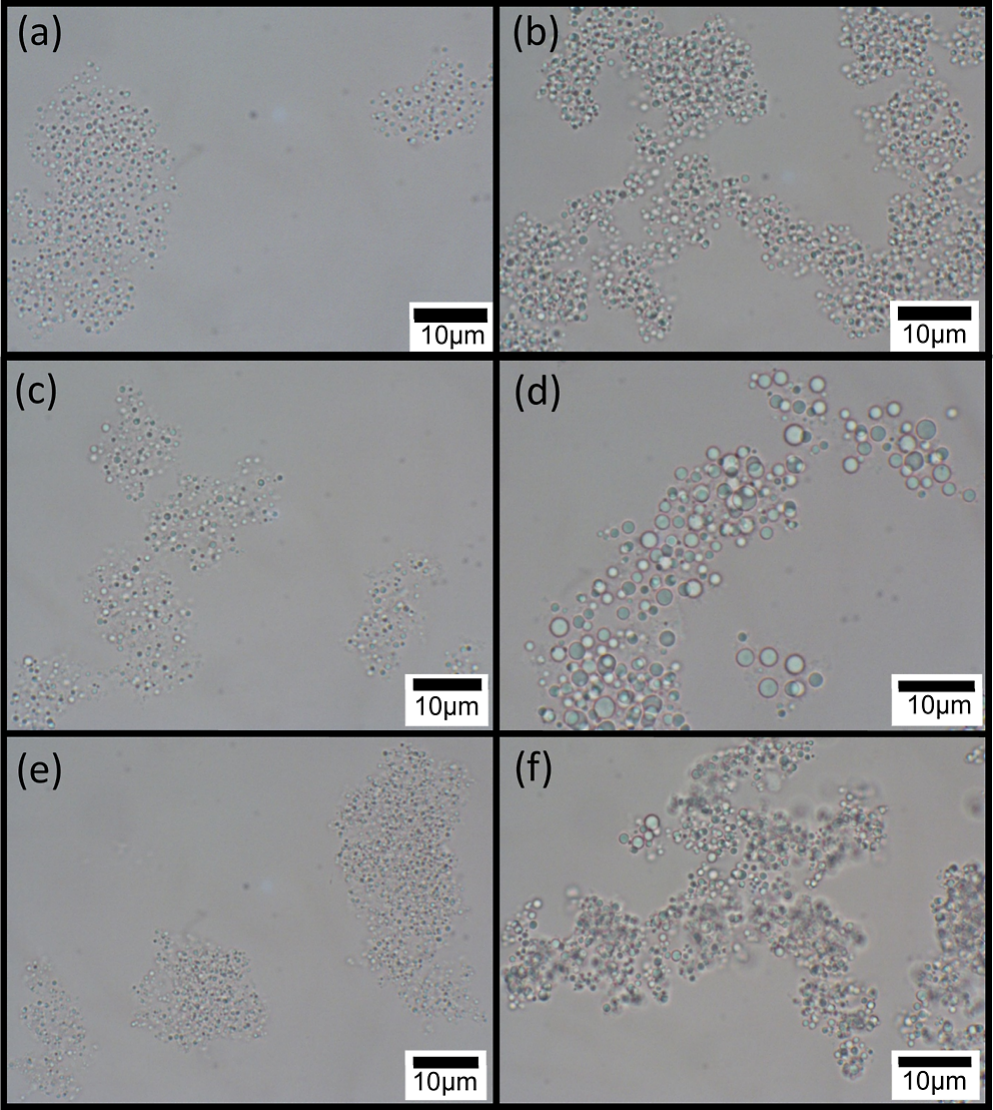
Light microscope images of cellulose-coated o/w emulsions fabricated with two cellulose/oil weight ratios and two homogenization pressures. (a) 1:1 cellulose/*n*-decane at 70 MPa; (b) 1:4 cellulose/*n*-decane at 70 MPa. (c) 1:1 cellulose/*n*-decane at 14 MPa; (d) 1:4 cellulose/*n*-decane at 14 MPa; (e) 1:1 cellulose/*n*-hexadecane at 14 MPa; and (f) 1:4 cellulose/*n*-hexadecane 14 MPa.

Cellulose-coated o/w emulsion droplets with n-decane in the core have a circular shape with clear visual borders at all cellulose/oil ratios and HPH pressures used. Figure 1 presents a wide distribution of the emulsion droplet dimensions in each kind of emulsion and each cellulose/oil ratio. Moreover, the droplet size with *n*-decane and *n*-hexadecane with a pressure homogenization of 14 MPa seems to be larger than the droplet size at a pressure homogenization of 70 MPa for both cellulose/oil ratios (Figure 1). The largest particles were achieved using *n*-decane as the hydrophobic phase with a cellulose/oil ratio of 1:4 at a homogenization pressure of 14 MPa. The cellulose-coated oil emulsion was also fabricated with triglycerides as the hydrophobic phases such as canola oil and castor oil. The structure of the emulsion droplets with canola oil and castor oil in the core also exhibits a circular shape with certain visual borders (Figure S1).

**Figure 2:**
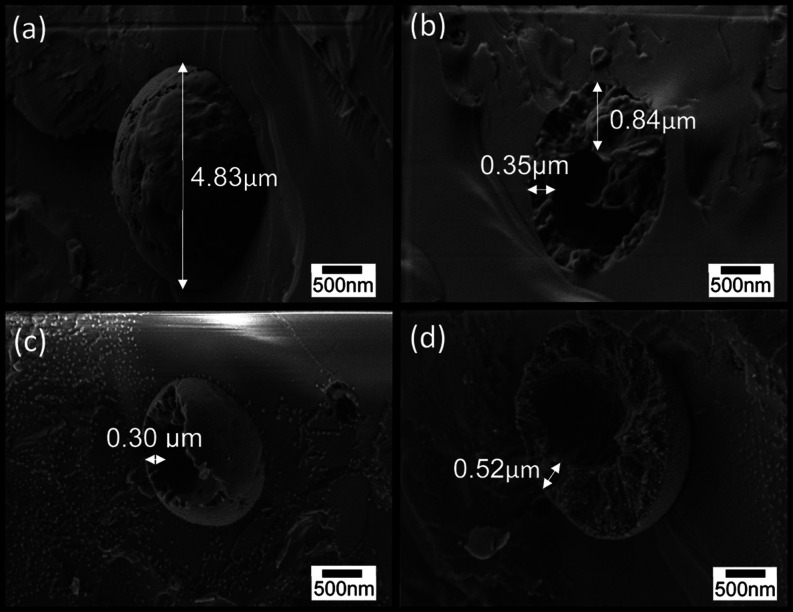
Cryo-SEM images of the fractured surface of vitrified cellulose-coated emulsion droplets, fabricated by HPH at 70 MPa. (a,b) Cellulose/*n*-decane wt ratio: 1:1; (c,d) cellulose/*n*-decane wt ratio: 1:4.

**Figure 3:**
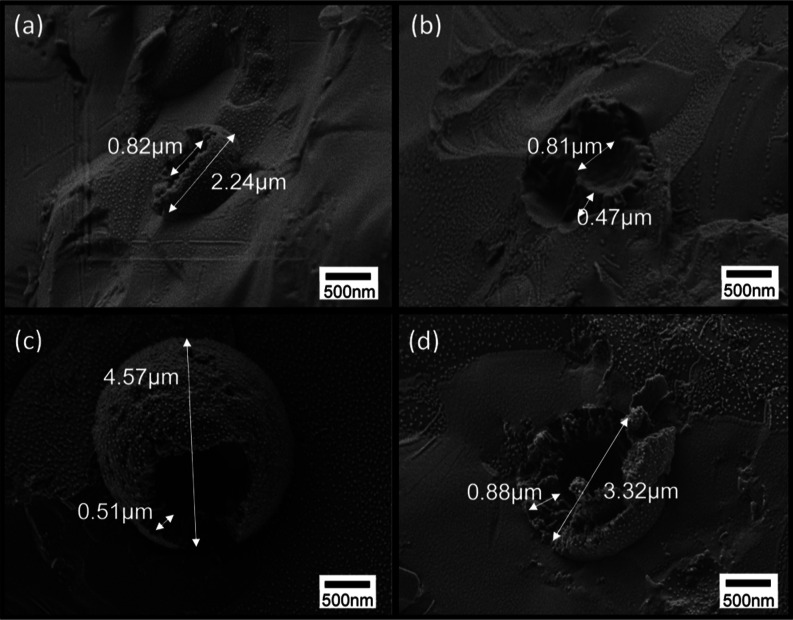
Cryo-SEM images of the fractured surface of vitrified cellulose-coated emulsion droplets, fabricated by HPH at 14 MPa. (a,b) Cellulose/*n*-decane wt ratio: 1:1; (c,d) cellulose/*n*-decane wt ratio: 1:4.

**Figure 4:**
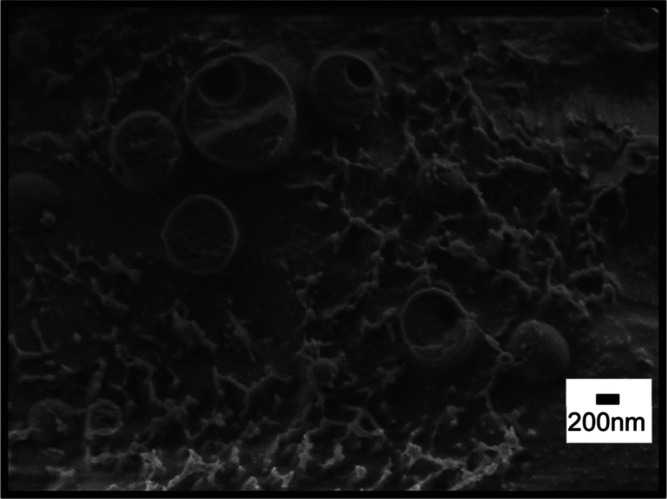
Cryo-SEM images of the fractured surface of vitrified cellulose-coated emulsion droplets, fabricated by HPH at 70 Mpa. Cellulose/canola oil ratio, 1:4.

Cryo-SEM imaging of the fractured surface of specimens also characterizes the structure of the emulsion droplets. Images of a cryo-fractured surface of rapidly frozen cellulose-coated *n*-decane micron-sized emulsion droplets in water fabricated at 70 and 14 MPa, after some sublimation of the surrounding water, are shown in Figures 2 and 3, respectively. The images reveal the internal structure of the fractured emulsion droplets and core of n-decane surrounded by a thick internal cellulose shell. The presence of the cellulose hydrogel at the external shell of the emulsion droplets is demonstrated in Figure S2 of the Supporting Information, using an ESB (energy selective back scattered) detector. Imaging contrast using this detector, based on the elemental composition of the specimen, allows us to distinguish between the cellulose hydrogel (brighter areas) and oil at the core (darker areas). Figure 2a exhibits an intact portion of the external shell, indicating that it is rather continuous and homogenous. In other cases, such as in Figures 2b–d and 3, apparently, the inner oil core was removed during fracture of the frozen samples, thus revealing the inner structure. A similar observation of cellulose-coated emulsion droplets made from cellulose solution in an aqueous solution of 8 wt % NaOH/6 wt % thiourea was obtained.^5^ The current study reveals a thick internal shell composed of porous cellulose, encompassing the space from which the oil core was removed, and which is surrounded by an apparently denser and more homogeneous external cellulose shell. The small spots in the images, appearing in some regions both of the particles and background, are assumed to be ice nanocrystals redeposited during some instability in the sublimation process. The droplet diameter ranges from 1 to 5 μm. The droplet dimension (Figure 3d) evaluated by cryo-SEM is in accordance with the light microscopy images which exhibit emulsion droplets with an average diameter of 3 μm. In the low-resolution image, Figure 4, small droplets can be seen with much free cellulose hydrogel. This indicates that not all the hydrogel was utilized in effective encapsulation of the emulsified oil. The fractured surface shown in Figure 4 reveals the inner structure of the canola oil emulsions. It exhibits the hydrophobic core canola oil, as in the case of smaller particles; apparently the fracture process cut through the frozen oil core. As discussed above, the oil core appears to be surrounded by a very thick internal cellulose shell. Moreover, the external cellulose shell surface is rather smooth, continuous, and homogeneous without any particle stabilization on the droplet surface. In some cases, an inner void appears in the frozen oil. We assume this to be an artifact of the rapid freezing process due to volume contraction of the oil core, being strongly attached to the encapsulating shell.

The images of the external cellulose shell surfaces appear to be rather smooth, homogeneous, and continuous, without any noticeable particles on the droplet surface, as would have been seen in particle-stabilized Pickering emulsions, such as those shown by Capron et al.^21^ for emulsions stabilized by cellulose nanocrystals. Costa et al.^9^ discussed the different methods of using solutions of unmodified cellulose for emulsion stabilization: by regeneration of a metastable dispersion of oil droplets in the cellulose solution^5,6^ and by homogenization of oil with dispersion of cellulose hydrogel microparticles regenerated from solution,^7,12,27^ the method employed in this article. In their evaluation, Costa et al.^9^ indicated emulsion droplets formed by the former method are stabilized by a smooth and continuous external cellulose shell, and the latter method results in emulsion droplets stabilized by the Pickering mechanism due to the adsorbed hydrogel particles, augmented by a viscous polymer network in the continuous medium. The observations reported here show, on the contrary, that both methods yield a non-particulate external cellulose shell that is rather smooth and homogeneous. Moreover, our studies show that the morphology in both cases is more complex, exhibiting a double-shell structure. The mechanism by which the regenerated cellulose hydrogel particles form a continuous coating on the hydrophobic emulsion droplets during the HPH process is not clear. One possibility is that this follows the Pickering-type emulsification discussed above and is due to the ensuing high shear forces. Furthermore, involvement of some hydrophobic hydrogel surfaces may be relevant, even if they exist as a minority. Existence of the hydrophobic surface in regenerated cellulose has been shown in different systems, such as when different coagulants are used.^28−30^

**Figure 5:**
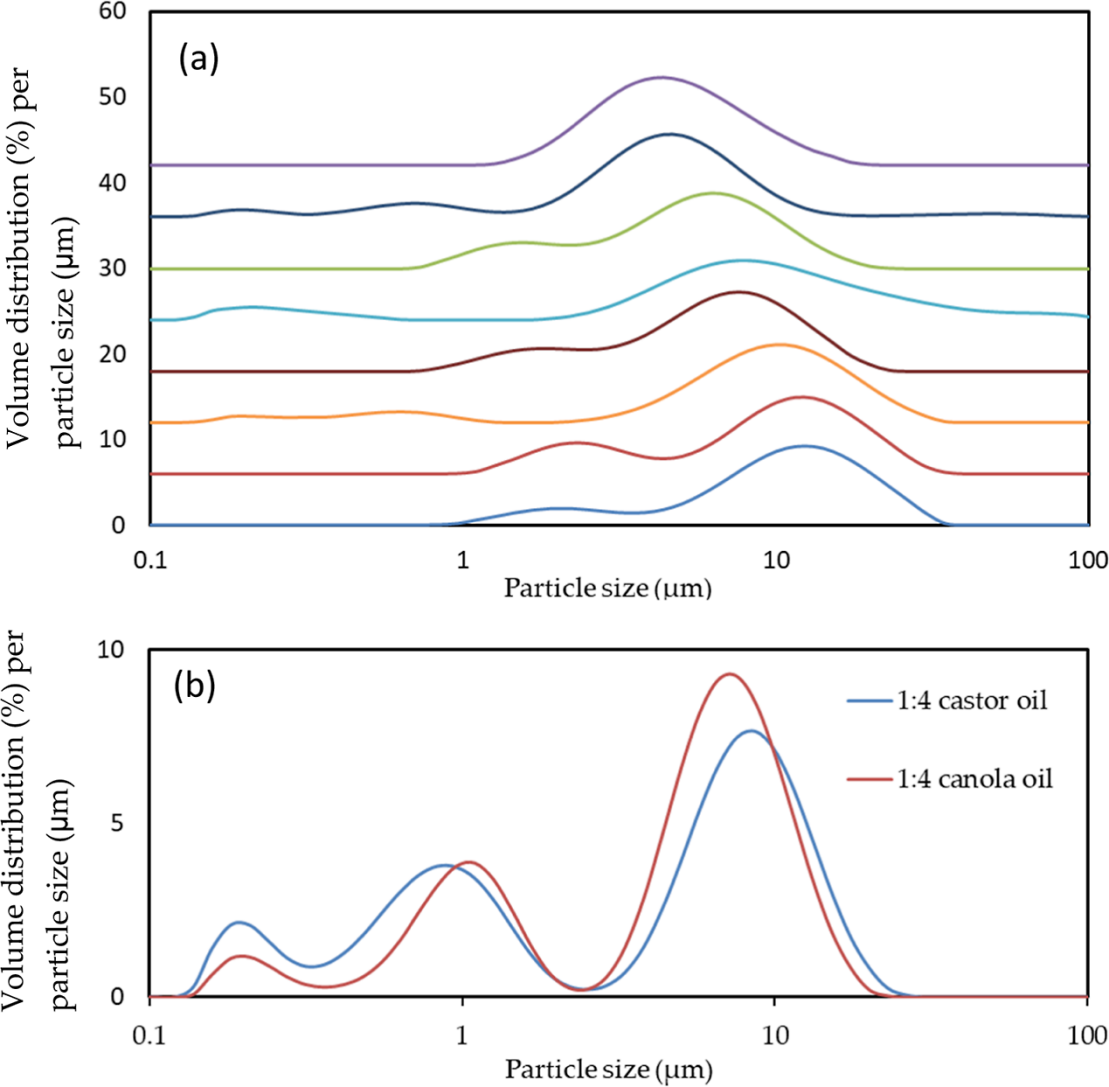
Particle size distribution by the volume evaluated by LS from cellulose-coated emulsions of (from bottom to top) (a) 1:1 decane 70, 1:4 decane 70, 1:4 hexadecane 70, 1:4 hexadecane 14, 1:1 hexadecane 70, 1:1 decane 14, 1:1 hexadecane 14, and 1:4 decane 14 and (b) 1:4 castor 70 and 1:4 canola 70.

Figure 5 presents LS measurements implemented on the cellulose-coated o/w emulsion with *n*-decane, *n*-hexadecane, and triacyl-glyceryl (TAG) in the core. The first peak at a lower particle size is attributed to individual emulsion droplets, while the second peak at a larger size is attributed to aggregation of these emulsion droplets. The droplet size with *n*-decane in the core (cellulose/oil 1:4, HPH at 14 MPa) exhibits the largest particle size. The measurements shown in Figure 5 are in good correlation with the light microscope images. Light microscope images in Figure 1 validate that the second peak is indeed attributed to aggregated emulsion droplets. It is evident that emulsifying the TAGs yields smaller-sized particles. This may be attributed to their low interfacial surface tension against water (21 and 31.5 mN/m for castor oil and canola oil, respectively),^31−33^ compared to the hydrocarbons (53.5 mN/m for hexadecane/water),^34^ which facilitates break-up into smaller droplets during emulsion fabrication.

**Figure 6:**
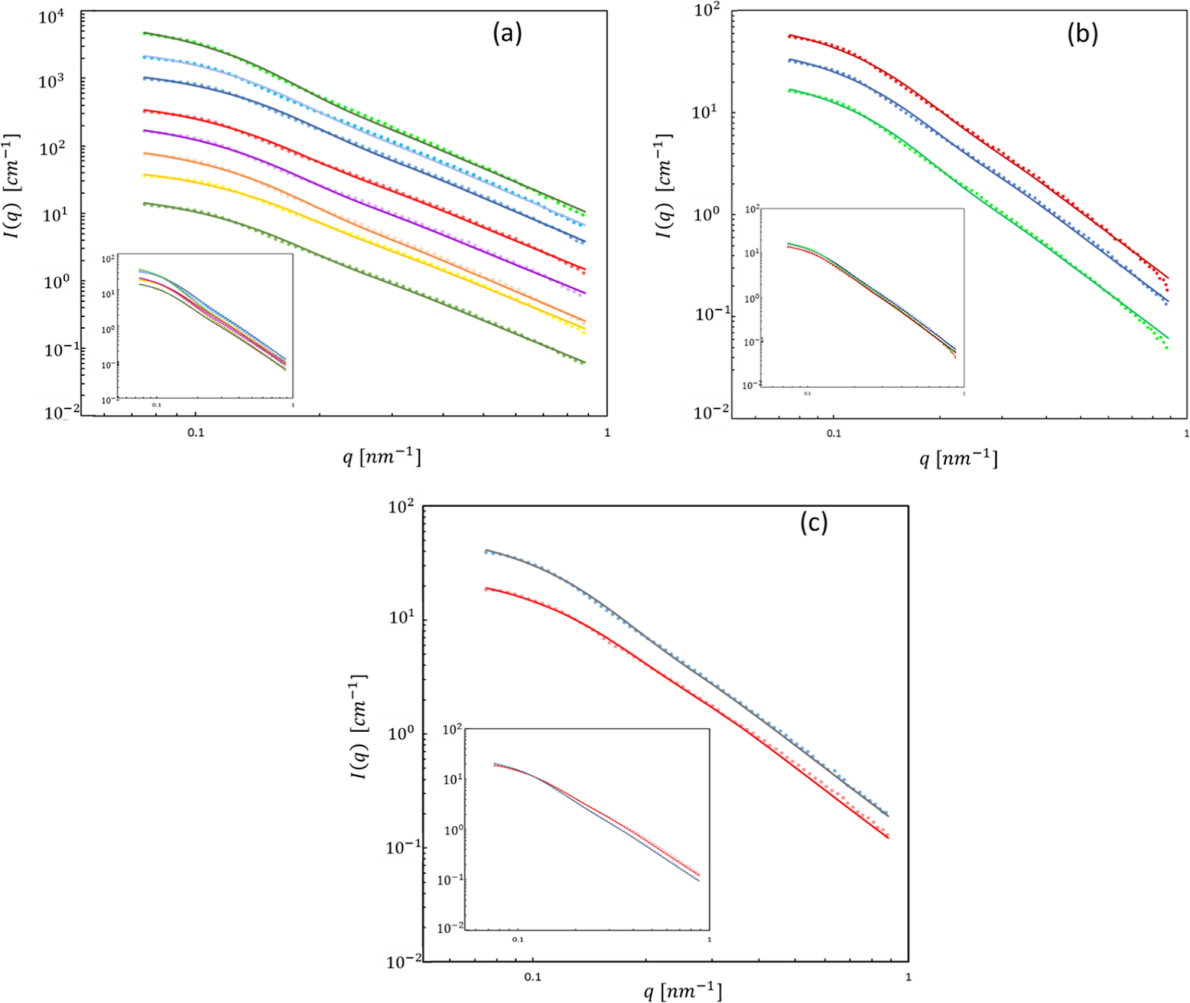
SAXS measurements (after background subtraction) and model fits for cellulose-coated emulsions of (from bottom to top) (a) 1:4 decane 14, 1:1 decane 14, 1:4 decane 70, 1:4 hexadecane 14, 1:1 hexadecane 14, 1:1 decane 70, 1:1 hexadecane 70, and 1:4 hexadecane 70 and (b) 1:4 castor 70, 1:1 castor 70, and 1:4 canola 70; each curve in Figure 6a,b is shifted vertically by factors of 2, so the scattering patterns do not overlap (the patterns of 1:4 decane 14 in (a) and 1:4 castor 70 in (b) are in the original scale). (c) Model fit for SAXS measurements of the cellulose hydrogel. Red and blue colors represent mechanical homogenization and HPH at 70 MPa, respectively. Solid line-measurement; dotted line-fit of the unified model. The inset shows the curves without shifting.

SAXS measurements were performed on the cellulose hydrogel microparticle dispersions and cellulose-coated particle emulsions fabricated using two homogenization pressures and two cellulose/oil ratios, with *n*-decane, *n*-hexadecane, and TAGs in the core. The measured data, after background subtraction, are shown in Figure 6. The main issue to be considered is to identify and model the source of the measured signal. First, we consider gross models of the particle shape. For this, a simple core–shell sphere and a spherical core with two shells were obtained as an initial approximation (eqs S1 and S2). This model calculation, using reasonable structural parameters estimated from the microscopy images, depicts the intensity of the scattering pattern in absolute units to be reduced to a negligible value at the measured *q*-range, below the noise level (Figure S3). This is due to the large gross dimensions of the emulsion particles in this work, diameter in the range of 0.1–4 μm, and shell thickness larger than 0.2 μm, being larger than the inverse of the smallest measurable *q*. Thus, we assume that the structure of the porous inner shell of the cellulose-coated o/w emulsion particles is the structural element that is responsible for the observed intensity at the measurable *q*-range.

Considering the intensity scattered from a dilute suspension of non-interacting particles with an inhomogeneous inner structure, *I*(*q*), it is useful to use its decomposition to a sum of three functions, developed by Stuhrmann for use in contrast-variation experiments, where the difference between the scattering length density of the particle and its surrounding medium (Δρ) is varied usually by changing the SLD of the medium^35,36^
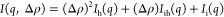
1where *I*_h_(*q*) is the homogeneous part, which is related to the particle shape, *I*_i_(*q*) is the heterogeneous part, which is related to the structure of inner inhomogeneities, and *I*_ih_(*q*) is a cross-term related to the coupling of scattering amplitudes of the overall shape and the inner inhomogeneities. Bianco et al. applied this relation to scattering from spherical particles with an inner random two-phase structure, whereby the size of the inhomogeneities is much smaller than the particle dimensions, showing that the second term (the cross-term) is negligible in this case.^37^ Seelenmeyer et al. used a similar two-term equation to analyze the scattering from core–shell emulsion particles having a solid polymer core surrounded by a shell of polymer hydrogel.^38^ Having established that the homogeneous term does not contribute significantly to the scattering signal in the measured *q*-range due to the large key dimensions of the particles, the scattering measurements are analyzed as due predominantly to the heterogeneous part: the porous cellulose shell. The small-scale inhomogeneities are considered to be due to the porous structure of the regenerated cellulose hydrogel comprising the inner shell. The measured scattering patterns shown in Figure 6 exhibit nearly a power-law dependence of intensity on the scattering vector, with an exponent of about 2.5. This indicates a possible fractal nature of the porous cellulose shell structure. A useful model for obtaining structural parameters of porous networks with some aspects of a fractal structure is the empirical Unified Guinier-exponential/power-law fit method developed by Beaucage.^39^ It applies multiple sets of Guinier and Power law equations to approximate the scattering from complex morphologies over a wide range of *q*. The mass fractal morphology was characterized by the unified equation for one structural level, that is, one set of Guinier and power law relations (eqs 2 and 3).^39^
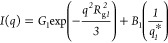
2
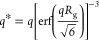
3where *i* is defined as the level number to describe structural characteristics at different size scales. *G* and *B* are prefactors related to different components of the structure, *R*_g_ is the radius of gyration of the compact structural component, and *P* is a power-law exponent characterizing the fractal aspect of the structure. 3 < *P* < 4 indicates a surface fractal which may be related to surface roughness of the inner structure (*P* = 4 indicates a smooth surface described the Porod relation), whereas *P* < 3 may indicate a mass-fractal nature of the aggregated structural components.^40^

As shown in Figure 6, all the SAXS patterns can be fitted by the Beaucage Unified model of level one. Table 1 summarizes the fitting results for cellulose-coated o/w emulsion droplets with *n*-decane, *n*-hexadecane castor oil, and canola oil in the core, as well as cellulose hydrogel particle suspensions. The hydrogel samples have the same cellulose content as the emulsions and were fabricated by the same dissolution-coagulation method, followed by either the mechanical homogenization alone or with subsequent HPH, at conditions used for the emulsion preparation (the complete table of fitted parameters is provided in the Supporting Information Table S1). The structural parameters obtained by fitting the Beaucage Unified model (radii of gyration and fractal dimensions) are similar for all emulsion and hydrogel samples studied. From this, we deduce that the inner shell structure of the cellulose-coated emulsion droplets does not depend on the emulsification process and its parameters (the cellulose: oil ratio, the oil type, and homogenization pressure).

**Table 1:** Parameters Obtained From the SAXS Measurements by Fitting the Beaucage Unified Model and Invariant Analysis

sample cellulose/oil wt. ratio, core liquid, HPH pressure MPa	*R*_g1_ (Å)	*P*_1_	*Q* ( × 10^–4^ cm^–1^ Å^3^)	% cellulose
1:1 decane 70	144	2.60	1.84	18
1:4 decane 70	153	2.60	0.95	58
1:1 decane 14	142	2.50	1.21	46
1:4 decane 14	151	2.50	0.74	67
1:1 hexadecane 70	154	2.60	1.59	30
1:4 hexadecane 70	167	2.60	1.26	44
1:1 hexadecane 14	151	2.50	1.26	44
1:4 hexadecane 14	156	2.50	1.12	50
1:1 castor 70	146	2.60	0.92	59
1:4 castor 70	146	2.60	0.86	62
1:4 canola 70	149	2.60	0.96	57
hydrogel mechanical homognizer	143	2.50	1.55	31
hydrogel HPH 70	153	2.50	1.21	46

The volume fraction of cellulose within the shells was estimated by the invariant analysis. The scattering invariant (*Q*_meas_) is the total integrated scattering intensity over the entire reciprocal space.^41^
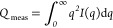
4

The Guinier function was used for low *q* extrapolation and Porod function for high *q* extrapolation.^42^ In general, for a two-phase system, the invariant may be related to the sample composition, irrespective of the structural details, as given by:^42^
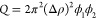
5where Δρ = ρ_1_ – ρ_2_ is the difference in scattering length densities (SLDs) between the two phases, and ϕ_1_ and ϕ_2_ are their respective volume fractions. Equation 5 is valid when the intensity is normalized to units of the scattering cross-section per unit volume. If the structural element responsible for the measured intensity occupies only a fraction of the sample volume under study, denoted by a volume fraction *f*, eq 5 should be modified according to:
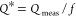
6since the measured intensity in absolute units (scattering cross-section squared) has been normalized to the total irradiated volume, not the volume of the sample responsible for the measured signal. Proceeding with our assumption that the measured scattering is only due to the structure of the porous inner shell, the invariant calculation can allow estimation of the volume fraction of cellulose in the porous cellulose hydrogel, making up the inner shell of the emulsion particles, denoted as ϕ_cell_ (or water: 1 – ϕ_cell_). For this, we need to assume further that the hydrogel in the inner shell has the same cellulose volume fraction as that of the free hydrogel in the aqueous medium. Alternatively, we can neglect the amount of free cellulose hydrogel particles. In the latter case, the calculated value of ϕ_cell_ is an upper limit. In either case, we can proceed by knowing the total volume fraction of cellulose in the sample, from the method of preparation, to be about 0.0067 (S3). By a simple volume balance, *f*ϕ_cell_ = 0.0067. Thus, using eq 6 and eq 5, the volume fraction of cellulose within the porous cellulose hydrogel comprising the inner shell can be estimated from the measured invariant, as:
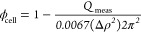
7

The invariant and the volume fraction of cellulose within the shell for all cellulose-coated emulsion samples are reported in Table 1. Following the SANS analysis of cellulose-coated emulsion droplets fabricated from ionic liquid solution,^6^ which is demonstrated by contrast variation measurements that the liquid within the porous cellulose inner shell is water, we consider the SLD’s for the calculation, using eq 7 as 1.45 × 10^–5^ and 9.47 × 10^–6^ Å^–2^ for cellulose and water, respectively, using their mass densities as 1.5 and 1.0 gr/cm^3^, respectively. According to Table 1, the inner shell of the cellulose-coated emulsion droplets contains cellulose ranges from about 20 to 60 vol %, much higher than those of the low cellulose volume fraction (∼3%) evaluated for the cellulose-coated emulsion droplets fabricated from ionic liquid solution.^6^ In addition, it can be seen that the cellulose volume fraction of the hydrogel is higher after HPH, indicating that some compaction of the hydrogel occurs during high-pressure processing. These observations seem to be in accordance with the images shown in Figures 2 and 3, which exhibit a solid yet porous inner shell between the inner core and the external thin homogenous cellulose coating. However, correlation of the cellulose content of the inner hydrogel shell of the emulsion droplets to the processing parameters cannot be elucidated from these measurements, and further studies are needed.

**Figure 7:**
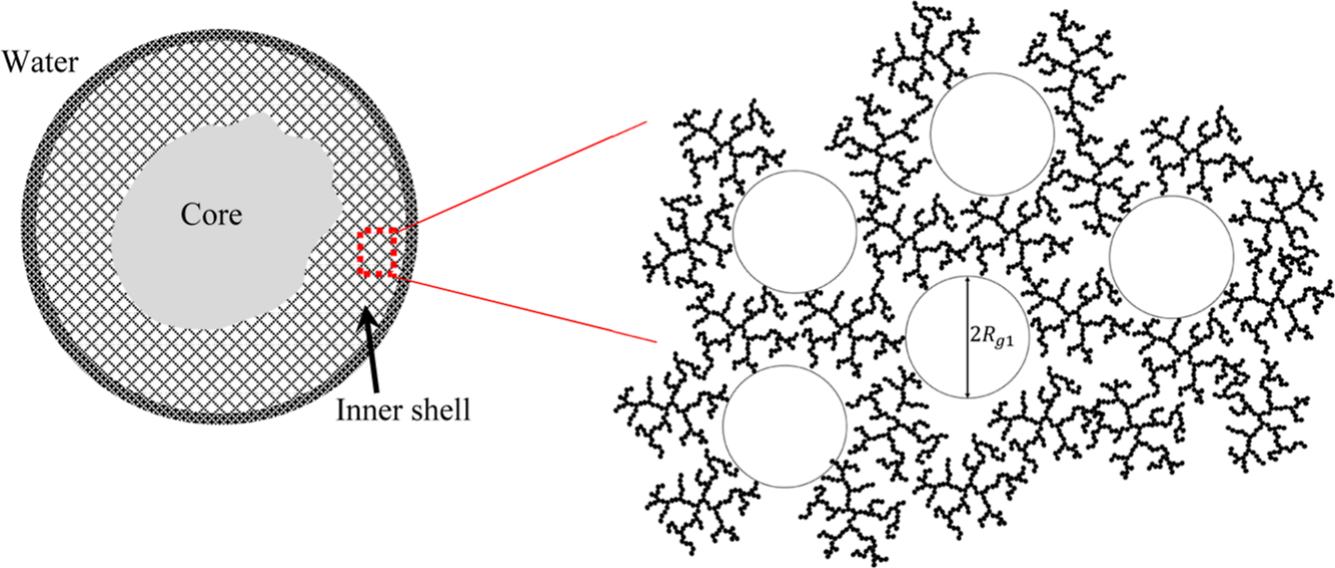
Scheme of the suggested structure of the cellulose-coated emulsion inner shell, according to SAXS measurements and invariant analysis.

A scheme of a suggested structure for the inner shell of the emulsion droplet is presented in Figure 7. It indicates that the porous hydrogel is inhomogeneous, consisting of pores, the size of which is a few tens of nanometers, embedded in a more dense highly branched hydrogel with an apparent fractal structure, suggestive of the cellulose aggregation process that occurs during regeneration from solution. This distinctive structure of cellulose-coated emulsion droplets can establish an innovative bio-reactor for one-pot processes for cellulose valorization. This process combines a cascade of reactions. One can envision cellulytic enzymes attached to the high surface-area emulsion droplets transforming the readily accessible porous cellulose into sugars or alcohols which can react with TAGs in the droplet core by *trans*-esterification catalyzed by lipase at the inner oil-hydrogel interface. The confined hydrogel layer provides an effective micro-environment for enzyme activity, isolated from the external aqueous medium. Furthermore, integration of emulsion droplets with engineered yeast provides a pathway for simultaneous saccharification and fermentation on a micron-scale, thus offering close contact at micron-scale dimensions, between all components, which is expected to facilitate mass transformation of products and reactants, with a negligible loss to the surrounding medium.

## Conclusions

In this research, the unique structure of oil in water (o/w) emulsions coated by unmodified cellulose was examined. Altering the cellulose: oil ratio, the oil type, and the homogenization pressure influences the emulsion dimensions but not on the inner shell structure of these emulsions. The invariant analysis exhibits good correlation with the cryo-SEM images. The cellulose volume fraction of the inner shell of the emulsions ranges from 20 to 60%. Controlled characteristics of the external and internal shells (composition, thickness, and porosity) will allow significant achievement in cost-effective biofuel processing.

## Supporting Information Available

Light microscopy images of cellulose-coated o/w emulsions fabricated with two different oils; cryo-SEM images of the fractured surface of vitrified cellulose-coated emulsion droplets, with the Everhart–Thornley detector (SE2) and ESB (energy selective back scattered electrons) detector; fluorescence microscope images of cellulose-coated o/w emulsion droplets; equations of a simple core–shell sphere and a spherical core with two shells as an initial approximation; calculation of the total volume fraction of cellulose; SAXS patterns of the measured data of the 1:1 cellulose: hexadecane emulsion fabricated at 70 MPa and a calculated model for a homogenous core-two shell spherical structure; and detailed parameters obtained by fitting the Beaucage Unified model and invariant analysis (PDF)
