# Supplementary material for: Structural Insights into Cellulose-Coated Oil in Water Emulsions
Source: Langmuir. 2022 Sep 7;38(37):11171–9. doi: 10.1021/acs.langmuir.2c00947 (PMC9494939; doi:10.1021/acs.langmuir.2c00947)
Supplement: Supplementary file 1 — la2c00947_si_001.pdf [file la2c00947_si_001.pdf]

Supporting information

Structural Insights into Cellulose-Coated Oil in Water Emulsions

Ester Korkus Hamal[1], Gilad Alfassi[2], Rafail Khalfin[1], Dmitry M. Rein[1], Yachin Cohen[1]*

[1]Department of Chemical Engineering, Technion - Israel Institute of Technology, Haifa 3200003, Israel

[2] Department of Biotechnology Engineering, ORT Braude College, Karmiel 2161002 Israel.

* Corresponding author. E-mail address: sesterko@campus.technion.ac.il

No. of pages: 5

No. of Figures: 3

No. of Equations: 2

No. of Tables: 1

Additional figures, Equations and Table as mentioned in the text.

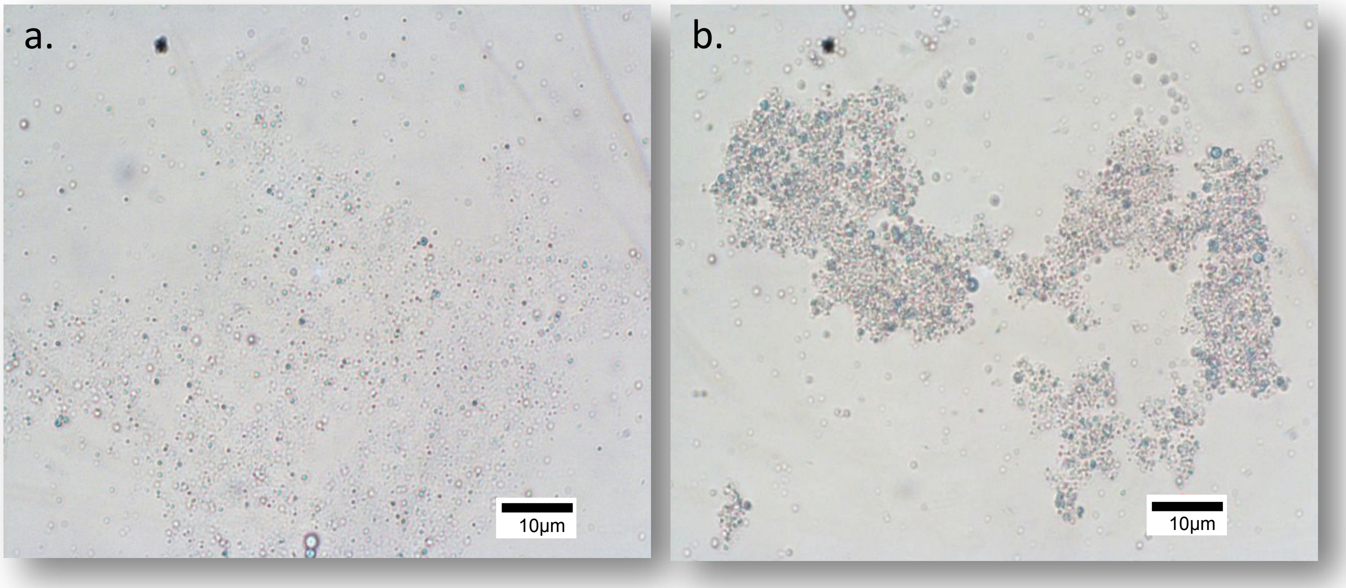

Figure S1. Light microscope images of cellulose-coated o/w emulsions fabricated with two different oils, high- pressure homogenization at 10,000 psi. a. cellulose:castor oil ratio: 1:4; b. cellulose:canola oil ratio: 1:4.

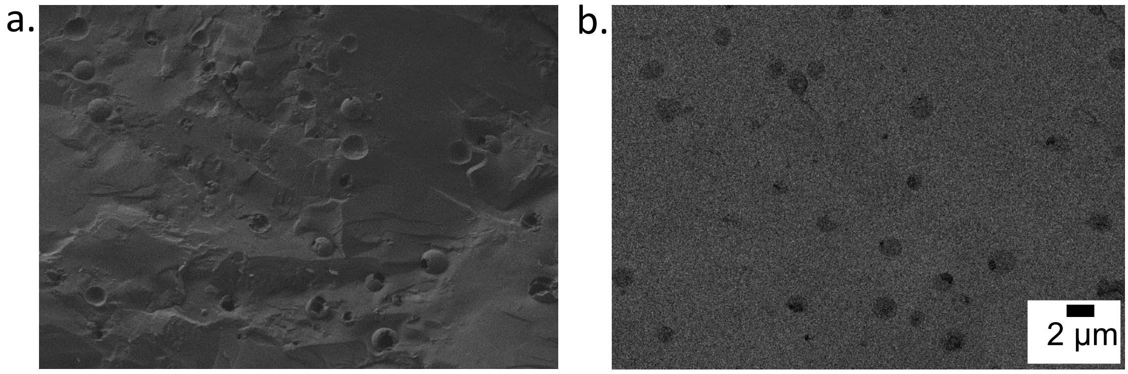

Figure S2. Cryo-SEM images of the fractured surface of vitrified cellulose-coated emulsion droplets, fabricated by HPH at 10,000 psi. cellulose:n-decane wt. ratio: 1:4. a. With the Everhart-Thornley detector (SE2), b. With ESB (energy selective back scattered electrons) detector.

Equation S1. The form factor of a spherical core shell

$$p(q) = [V_{sh}\rho_{sh}F_{sphere,sh} - V_{core}(\rho_{sh} - \rho_{core})F_{sphere,core}]^2$$

$$F_{sphere}(q,R) = 3\frac{\sin(qR) - qR\cos(qR)}{(qR)^3}$$

Equation S2. The form factor of a spherical core two shells

$$p(q) = [V_{core}(\rho_{core} - \rho_{sh,1})F_{sphere,core} + V_{sh,1}(\rho_{sh,1} - \rho_{sh,2})F_{sphere,sh,1} + V_{sh,2}(\rho_{sh,2} - \rho_{sol})F_{sphere,sh,2}]^2$$

Where V is the particle volume, ρ is the SLD, and R is the radius. R_{core} is the core radius, $R_{sh,1}$ is the radius of the inner shell with thickness d_1 ($R_{sh,1} = R_{core} + d_1$), and $R_{sh,2}$ is the radius of the outer shell with thickness d_2 ($R_{sh,2} = R_{core} + d_1 + d_2$).

S3. The total volume fraction of cellulose

For a sample of cellulose hydrogel dispersion containing 1 wt% cellulose

Using the mass density of cellulose as 1.5 gr/cm^3

Thus, the total volume fraction of cellulose in the sample is: $\frac{0.01}{1.5} = 0.0067$

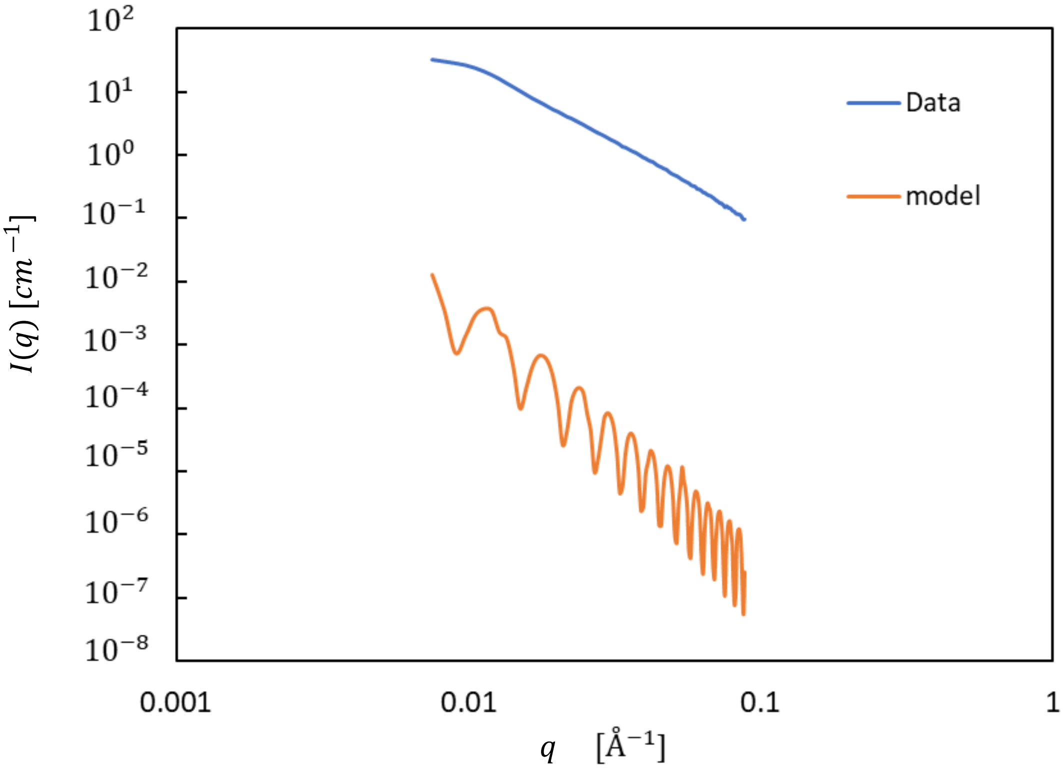

Figure S3. SAXS patterns: at top the measured data of the 1:1 cellulose: hexadecane emulsion fabricated at 70 MPa (after background subtraction) and at bottom a calculated model for a homogenous core-two shells spherical structure with the particle concentration approximated from the emulsion composition, and core radius and shell thicknesses approximated from SEM images. Both curves are plotted in absolute units, using dimensions estimated from microscope images and the known cellulose content. The comparison shows the intensity measured in the accessible range of scattering vectors must be representative of structures smaller than the shell-thickness, interpreted to be due to the gel structure of the inner shell.

Table S1. Parameters obtained from Beaucage Unified model and invariant analysis.

**Sample** Cellulose:oil wt. ratio, core liquid, HPH pressure psi	R_{g1} (Å)	P_1	$B_1(\times10^{-4} cm^{-1})$	$G_1(cm^{-1})$	Q $(\times10^{-4} cm^{-1}$Å$^3)$	**%cellulose**
1:1 decane 10,000	144	2.60	2.14	46.4	1.84	18
1:4 decane 10,000	153	2.60	1.09	28.7	0.95	58
1:1 decane 2,000	142	2.50	2.14	26.3	1.21	46
1:4 decane 2,000	151	2.50	1.27	19.0	0.74	67
1:1 hexadecane 10,000	154	2.60	1.83	49.6	1.59	30
1:4 hexadecane 10,000	167	2.60	1.46	59.9	1.26	44
1:1 hexadecane 2,000	151	2.50	2.08	31.1	1.26	44
1:4 hexadecane 2,000	156	2.50	1.83	32.3	1.12	50
1:1 castor 10,000	146	2.60	1.15	22.2	0.92	59
1:4 castor 10,000	146	2.60	1.02	19.9	0.86	62
1:4 canola 10,000	149	2.60	1.18	26.8	0.96	57
Hydrogel mechanical homognizer	143	2.50	2.75	27.8	1.55	31
Hydrogel HPH	153	2.50	2.16	31.5	1.21	46
